# Out of Context, Beyond the Face: Neuroanatomical Pathways of Emotional Face-Body Language Integration in Adolescent Offenders

**DOI:** 10.3389/fnbeh.2019.00034

**Published:** 2019-02-26

**Authors:** Hernando Santamaría-García, Agustin Ibáñez, Synella Montaño, Adolfo M. García, Michel Patiño-Saenz, Claudia Idarraga, Mariana Pino, Sandra Baez

**Affiliations:** ^1^Departamentos de Psiquiatría y Fisiología, Pontificia Universidad Javeriana, Bogotá, Colombia; ^2^Centro de memoria y cognición Intellectus, Hospital Universitario San Ignacio, Bogotá, Colombia; ^3^Grupo de Investigación Cerebro y Cognición Social, Bogotá, Colombia; ^4^Laboratory of Experimental Psychology and Neuroscience (LPEN), Institute of Cognitive and Translational Neuroscience (INCYT), INECO Foundation, Favaloro University, Buenos Aires, Argentina; ^5^National Scientific and Technical Research Council (CONICET), Buenos Aires, Argentina; ^6^Departamento de Psicología, Universidad Autónoma del Caribe, Barranquilla, Colombia; ^7^Center for Social and Cognitive Neuroscience (CSCN), School of Psychology, Universidad Adolfo Ibáñez, Santiago de Chile, Chile; ^8^Australian Research Council Centre of Excellence in Cognition and its Disorders, Sydney, NSW, Australia; ^9^Faculty of Education, National University of Cuyo (UNCuyo), Mendoza, Argentina; ^10^ Departamento de Psicología, Universidad de los Andes, Bogotá, Colombia; ^11^Departamento de Psicología, Universidad de la Costa, Barranquilla, Colombia

**Keywords:** adolescent offenders, emotion recognition, emotion integration, brain morphology, disruptive behaviors

## Abstract

**Background**: Adolescent offenders (AOs) are characterized by social-norm transgression and aggressive behaviors. Those traits have been associated with alterations in socio-cognitive processes, including facial emotion recognition. While this would suggest that AOs tend to interpret negative emotional cues as threatening information, most research has relied on context-free stimuli, thus failing to directly track integrative processes typical of everyday cognition.

**Methods**: In this study, we assessed the impact of body language and surrounding context on facial emotion recognition in AOs and non-offenders (NOs). We recruited 35 AOs from a reform school for young male offenders and 30 NOs matched for age and sex with the former group. All participants completed a well-validated task aimed to determine how contextual cues (i.e., emotional body language and surrounding context) influence facial emotion recognition through the use of congruent and incongruent combinations of facial and bodily emotional information.

**Results**: This study showed that AOs tend to overvalue bodily and contextual signals in emotion recognition, with poorer facial-emotion categorization and increased sensitivity to context information in incongruent face-body scenarios. This pattern was associated with executive dysfunctions and disruptive behaviors, as well as with gray matter (GM) of brain regions supporting body-face recognition [fusiform gyrus (FG)], emotion processing [cingulate cortex (CC), superior temporal gyrus (STG)], contextual integration (precuneus, STG), and motor resonance [cerebellum, supplementary motor area (SMA)].

**Discussion**: Together, our results pave the way for a better understanding of the neurocognitive association between contextual emotion recognition, behavioral regulation, cognitive control, and externalized behaviors in AOs.

## Introduction

The term “adolescent offenders” (AOs) refers to subjects below 18 years of age who disrupt social and legal regulations and manifest delinquent behavior, ranging from minor offenses (such as underage smoking/drinking) to property crimes and violent crimes (Dodge et al., 1990; Philipp-Wiegmann et al., 2017). These individuals usually transgress social norms and exhibit antisocial and aggressive conduct (Gonzalez-Gadea et al., 2014; Piotrowska et al., 2015; Piquero et al., 2015). This pattern of disruptive behaviors can be understood as a general maladjustment to the immediate social context. For instance, disorderly conduct in AOs is associated to reduced abilities in different socio-cognitive functions, crucially including emotion recognition (Fairchild et al., 2009; Sato et al., 2009). This domain, which proves vital predicting social behavior (Frith, 2009; Ibáñez et al., 2014), entails highly context-dependent dynamic processes (Frith, 2009) that are sensitive to surrounding visual scenes (Barrett et al., 2011), verbal cues (Hassin et al., 2013), bodily signals (Aviezer et al., 2012), and other faces (Oosterhof and Todorov, 2008). Although AOs have been shown to exhibit difficulties in this domain (Fairchild et al., 2009; Sato et al., 2009), most studies have employed isolated stimuli indexing recognition of decontextualized emotions, thus proving blind to how the phenomenon operates under more ecological circumstances. To bridge this gap, here we evaluated how contextual information (i.e., body language) modulates facial emotion recognition in AOs, also assessing neuroanatomical markers of this process.

Adolescence is considered a critical period for developing socio-cognitive and affective processes in response to social-context requirements (Burnett et al., 2011). Many of those processes appear to be affected in AOs, who exhibit abnormal performance in empathy (Gonzalez-Gadea et al., 2014), moral judgment (Stams et al., 2006), and social decision-making (van den Bos et al., 2014) tasks, alongside different types of dysfunctional and delinquent behaviors (Hubble et al., 2015; Piquero et al., 2015).

Critically, socio-cognitive and behavioral problems in AOs have also been associated to facial emotion recognition difficulties (Fairchild et al., 2008, 2009; Passamonti et al., 2010). In the same vein, adolescents with conduct disorders manifest impairments on behavioral and psychophysiological measures of emotional recognition function, including facial expression recognition (Fairchild et al., 2009) and fear conditioning (Fairchild et al., 2008). In addition, compared to controls, AOs exhibit increased amygdala activation in presence of faces with negative valence (Passamonti et al., 2010).

Two contrastive theories have linked aggression to emotion recognition in AOs. On the one hand, aggressive behaviors may reflect increased sensitivity towards hostile signals (e.g., stimuli conveying disgust or anger), leading to defensive and maladaptive behaviors (Dodge et al., 1990; Jusyte and Schonenberg, 2017). This hostile misattribution bias in adult samples has been associated to reactive behaviors, including neural modulations in action preparation areas and physiological reactions related to stress (de Gelder et al., 2004; Grèzes et al., 2007; Fairchild et al., 2008, 2009). One the other hand, a reduced ability in recognizing social signals of distress (for instance the recognition of sad or fear emotions in others) may also be associated to disruptive behaviors observed in AOs (Blair et al., 2001; Bowen et al., 2014). Arguably, a decreased ability in negative emotions lead to offenders to ignore alarm or suffering cues in others and it could increase a non-prososcial behavior (Blair et al., 2001). The evidence suggests that both factors may be operative in the distinctive pattern observed in AOs.

Facial emotion recognition rests on the implicit integration of interoceptive signals and features external to facial expressions proper, including body language (de Gelder et al., 2004; Aviezer et al., 2012), voices (de Gelder et al., 1999; Davies-Thompson et al., 2018), verbal descriptions (Ferrari et al., 2016), visual scenes (Van den Stock et al., 2014; Wieser and Keil, 2014), and other forms of contextual information (Aviezer et al., 2012; Hassin et al., 2013; Couto et al., 2015; Adolfi et al., 2016). The perception of body signals and the surrounding context is highly relevant for unveiling emotional integration (de Gelder, 2006), with bodily cues affecting emotion recognition more than faces that express intense emotions (Aviezer et al., 2012).

The implicit integration of emotional information involves the activity of different brain networks, including: (a) areas of the visual network, such as the primary visual cortex (V1), the lateral geniculate nucleus, and the superior colliculus (de Gelder, 2006; Tamietto and de Gelder, 2010; Bachmann et al., 2018); (b) regions subserving action observation and motion perception, such as the premotor cortices, the inferior frontal gyrus, the inferior parietal lobe, the extrastriate body area, the fusiform body area, and the superior temporal sulcus (de Gelder, 2006; Tamietto and de Gelder, 2010; Burra et al., 2017); (c) areas involved in perceiving social information, like the temporo-parietal junction, the medial prefrontal cortex, the precuneus, and the orbitofrontal cortex (de Gelder, 2006; Tamietto and de Gelder, 2010; Bachmann et al., 2018); and (d) regions implicated in emotional processing, including the orbitofrontal cortex, the anterior cingulate cortex (CC), the precuneus, the amygdala, hypothalamus, the basal ganglia, and the periaqueductal gray (Grèzes et al., 2007; Vytal and Hamann, 2010; Van den Stock et al., 2014; Diano et al., 2017; Bachmann et al., 2018).

By the same token, the integration of emotional and social information from different sources (e.g., conveyed through direct or indirect gazes in a social context) may rely on dissociable cellular and physiological substrates. In particular, congruent social signals (e.g., a direct gaze in a social context) are mainly integrated by magnocellular channels tuned to low spatial frequencies, whereas incongruent signals (e.g., indirect eye gaze in a social context) are more integrated in parvocellular channels and high spatial frequencies. Note, however, that low spatial frequency emotional signals are also processed non-consciously (Burra et al., 2017).

Despite its cross-dimensional nature, emotion recognition in AOs has been mainly studied through decontextualized photographs of isolated facial expressions (Fairchild et al., 2008, 2009; Passamonti et al., 2010) –for an exception, see the study by Gonzalez-Gadea et al. (2014), although no body language or neural markers were assessed. Importantly, since such atomistic tasks can be solved *via* explicit knowledge, they seem insufficient for capturing emotion processing as manifested in daily life (Aviezer et al., 2012; Gonzalez-Gadea et al., 2014).

To date, only a few studies have explored face-body integration processes, targeting only adult offenders. Most of these works have reported impaired facial emotion recognition and attentional biases towards angry body postures (Kret and de Gelder, 2013; Kuin et al., 2017). Violent offenders tend to exhibit a bias towards aggressive body language, including anger or disgust postures (Kret and de Gelder, 2013). Emotional recognition is also biased by specific types of violent behavior, with deficits in recognizing distressful cues (e.g., sadness or fear) proving more marked in reactive rather than proactive violent offenders (Philipp-Wiegmann et al., 2017).

Neurally speaking, performance in social and emotional tasks has been previously associated to the gray matter (GM) volume (Ashburner and Friston, 2000; Yue et al., 2016; Uono et al., 2017) and activity (Vytal and Hamann, 2010; Diano et al., 2017; Bachmann et al., 2018) of specific brain areas, including the ventral medial frontal cortices (implicated in the recognition of sadness), the inferior frontal gyrus (associated with anger and disgust), the superior temporal gyrus (STG; linked to recognition of happiness), the bilateral insula (a putative substrate of anger recognition and disgust), and the amygdala (putatively related to the recognition of fear; Vytal and Hamann, 2010; Diano et al., 2017; Bachmann et al., 2018).

Concerning the integration of contextual body signals in facial emotion perception, previous studies have shown an association with the GM volume of temporal areas—including the parahippocampus, the amygdala (Kumfor et al., 2018), and the fusiform gyrus (FG; Van den Stock et al., 2014; Kumfor et al., 2018)—as well as to regions implicated in the processing of emotional body language—i.e., the CC (Maier and di Pellegrino, 2012) and the precuneus (Ahmed et al., 2015). Also, emotion processing has been linked to motor-related regions, including the cerebellum (Kumfor et al., 2018; Poyo Solanas et al., 2018).

In addition, previous studies have shown that bodies convey not only emotional and social information, but also emotion-related action intentions (de Gelder et al., 2004, 2006; Van den Stock et al., 2014). A dissociable pattern of brain activation has been reported in response to emotional body postures. Particularly, happy bodily postures activate visual areas, including the middle occipital gyrus, the inferior temporal gyrus, and the intraparietal sulcus (Poyo Solanas et al., 2018). By contrast, the recognition of fearful body positions has been associated with activity in areas supporting emotional and social processing, like the posterior superior temporal sulcus, the ventral prefrontal cortex, and the amygdala (de Gelder et al., 2004; de Gelder, 2006; Vuilleumier and Driver, 2007). Also, recognition of fearful body postures has been related to motor resonance areas [e.g., supplementary motor area (SMA; Peelen et al., 2007)] and regions involved in the monitoring and representation of bodily states (i.e., insula; Karnath et al., 2005; Peelen and Downing, 2007; Peelen et al., 2007).

Against this background, and through a well-validated task (Aviezer et al., 2012; Kumfor et al., 2018), we aimed to investigate the integration of emotional information from faces and bodies with contextual information in a AOs compared to non-offenders (NOs). Our focus was on how contextual cues (i.e., emotional body language and surrounding context) influence facial emotion recognition in the former group. Additionally, we sought to establish key neuroanatomical correlates of such contextual integration.

We predicted that, compared to NOs, AOs would exhibit reduced ability to integrate bodily and facial emotional cues, alongside a major bias in perceiving the former. Considering that AOs exhibit difficulties in recognizing negative emotions (Blair et al., 2001; Bowen et al., 2014) and present a marked bias towards perceiving hostile scenarios (Kret and de Gelder, 2013), we hypothesized that they would be unable to properly recognize negative emotions while showing major sensitivity towards body signals that convey hostile positions (i.e., anger or disgust body positions). This bias should prove more salient in incongruent contexts, as those scenarios convey more conflicting body-face contextual information and require more robust integration mechanisms. We further surmised that these patterns would be related to levels of disorderly conduct (Bowen et al., 2014). Additionally, those difficulties should be related to the subjects’ executive functioning, considering that this domain is crucial for social adaptation in adolescents and represents a key determinant for the perception and integration of emotional cues (Escobar et al., 2014; Gonzalez-Gadea et al., 2014).

Furthermore, we predicted that contextual integration in AOs would be associated with GM volume in areas subserving context integration (i.e., temporal regions, body language areas; Ibañez and Manes, 2012; Baez and Ibanez, 2014; Baez et al., 2017a) and emotion recognition processes (frontal and cingulate regions and precuneus; Kumfor et al., 2018).

## Materials and Methods

### Participants

The study comprised 65 male participants, including 35 AOs and 30 NOs, matched for age (*F*_(1,64)_ = 0.26, *p* = 0.21). However, AOs and NOs differed in education level (*F*_(1,64)_ = 4.56, *p* < 0.05; see Table 1). AOs were recruited from a reform school for young male offenders in Barranquilla, Colombia. These subjects had been imprisoned for various reasons, including homicide attempt, homicide, theft, and illegal arm possession, among others (see Table 2). NOs were recruited from schools located in the same district of residence of AOs.

**Table 1:** Demographical and neuropsychological data comparison between offender adolescents (AOs) and non-offenders (NOs).

	Offender adolescents (*N* = 35)	Non-offenders (*N* = 30)	Significance level
**Demographical data**			
Age	17.22 (1.35)	16.9 (1.56)	*p* = 0.21
Education (years)	8.37 (2.05)	9.83 (1.14)	*p* < 0.05
**Neuropsychological data executive functions**			
Fluid intelligence (RSPM)	19.05 (3.49)	19.63 (3.42)	*p* = 0.56
Motor programming	3.0 (0.10)	2.86 (0.43)	*p* = 0.33
Conflicting instructions	2.85 (0.32)	2.83 (0.37)	*p* = 0.88
Verbal inhibitory control	3.74 (1.09)	5.16 (0.98)	*p* < 0.01
Abstraction (proverbs)	1.51 (0.81)	1.76 (0.56)	*p* = 0.24
Backward digit span	3.02 (1.15)	3.8 (1.27)	*p* < 0.01
Spatial working memory	2.17 (1.07)	2.66 (1.12)	*p* = 0.11
Go/no-go	2.71 (0.45)	2.9 (0.25)	*p* < 0.05
Total IFS score	20.74 (3.7)	23.2 (3.22)	*p* < 0.01

**Table 2:** Description of type of offenses in the offender adolescents group.

		Percentage of cases
Type of crime	Homicide attempt	5.7%
	Homicide	40%
	Theft (qualified or aggravated)	31.4%
	Illegal carrying of weapons	17.1%
	Extortion	2.9%
	Sexual related violence	2.9%

Recruitment was authorized and assisted by the schools’ principals and teachers. Inclusion criteria for control participants were: (a) gender (male); (b) age (between 15 and 18 years old); (c) education level (less than 12 years of education); and (d) absence of history of psychiatric or neurological disorders.

All participants completed a structured admission interview to rule out psychiatric disorders and ensure that they were not under pharmacological treatment during the assessment. All participants and parents/tutors provided written informed consent in agreement with the Helsinki declaration. The protocol was approved by the Ethics Committee of the Universidad Autónoma del Caribe.

### Instruments

#### Executive Functions

Participants completed a battery of tasks tapping executive functions (EFs) and fluid intelligence (FI). The former were assessed *via* the INECO Frontal Screening (IFS) test (Torralva et al., 2009), a sensitive tool that has been used in different clinical and non-clinical populations (Baez et al., 2014, 2017b; Nunes et al., 2014), including AOs (Gonzalez-Gadea et al., 2014). The IFS comprises eight subtasks tapping on motor programming, conflicting instructions, verbal inhibitory control, abstraction ability (proverbs interpretation), backward digit span, spatial working memory, and a go/no-go test. A mean total score is calculated from the sum of the subtask scores (30 points). A 25-point cutoff score has shown a sensitivity of 96.2% and a specificity of 91.5% in detecting executive impairments (Torralva et al., 2009). Relative to NOs, AOs showed significantly lower IFS scores (*F*_(1,64)_ = 8.0, *p* < 0.01). Comparisons for each subtask are reported in Table 1.

#### Fluid Intelligence Measure

FI was examined through Raven’s Standard Progressive Matrices (Raven, 1960). Following this procedure, participants completed a series of drawings by considering the spatial organization of an array of objects, identifying relevant features, and choosing one object that matched one or more of the identified features. In this study we used an adapted and validated local version of the battery (de Pedraza et al., 2012). No significant difference in FI was observed between groups (*F*_(1,64)_ = 0.45, *p* = 0.51).

#### Behavioral Disturbances

Levels of disorderly conduct in AOs were established by considering two independent indices: months of jail sentence [mean = 27.9 months; standard deviation (SD) = 2.2 months] and number of relapses (mean = 1.6 relapses; SD = 1.08) during the entire life of AOs. Types of delinquent behaviors in AOs are further described in Table 2. Participants in this group did not differ in terms EFs and education according to the type of offense (*F*_(5,34)_ = 2.16, *p* = 0.75).

#### Emotion Recognition Task

Participants completed two tasks based on reported stimulus sets (Aviezer et al., 2012; Kumfor et al., 2018).

##### Task A: Recognition of Context (Body Alone)

Participants viewed 40 pictures of bodies (with covered faces) belonging to four conditions (anger, disgust, fear, sadness), with 10 pictures per emotion. They were asked to point to the label that best described the emotion expressed through body language (“anger,” “disgust,” “fear,” “sadness”) and context information (some cues supporting body emotions, such as a knife accompanying a threatening body context).

##### Task B: Contextual Effects (Body and Face Assessment)

As in task A, participants were presented with pictures of male faces depicting four types of emotion (anger, disgust, fear, sadness) and pictures of male bodies depicting the same four types of emotions. Participants viewed 80 pictures of subjects in which a congruent (*n* = 40) or an incongruent (*n* = 40) context (i.e., emotional body language) accompanied the facial expression. Thus, the participants saw 10 congruent trials per emotion (face and body emotion coincided). Furthermore, the participants saw five incongruent trials for each incongruent face-body combination (e.g., face of anger with body of disgust, face of anger with body of fear, among other combinations). Participants were asked to point to the label that best matched the facial expression. Following previous procedures (Aviezer et al., 2012; Kumfor et al., 2018), performance was assessed *via* two indices: categorization accuracy and contextual influence. The former consisted in the mean of correctly labeled trials for both congruent and incongruent contexts. The latter was defined as the percentage of times the face was labeled as expressing the contextual emotion (e.g., when a disgust face in a fear context is labeled as expressing fear), as opposed to any other emotion. We calculated: (a) a measure for congruent context—identical to categorization accuracy for congruent trials; and (b) a measure for incongruent contexts—the degree to which contextual information (i.e., body language) affects facial emotion recognition.

**Figure 1:**
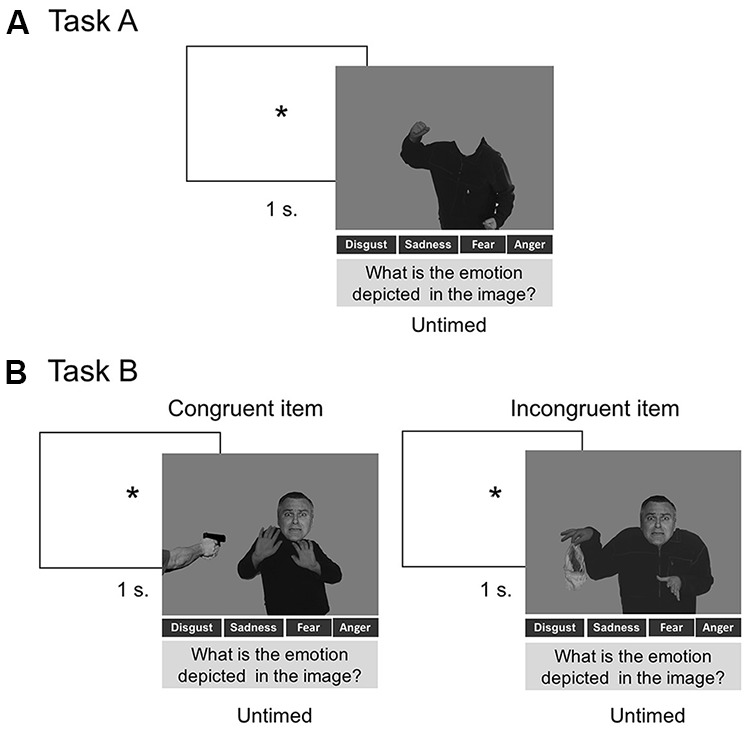
Panel **(A)** shows the procedure of Task A. Panel **(B)** shows the procedure used in Task B, including a trial with a congruent item and a trial with an incongruent item. The stimuli of this procedure were adapted from Aviezer et al. (2008). Reproduced with permission from Aviezer et al. (2008). *Indicates attention fixation signal.

For both tasks, images were randomized and presented one at a time with the emotional labels at the bottom of the screen, and they remained visible until a response was made. No time limit was set for responding and no feedback was provided (Figure 1).

### MRI Scanning

A subsample of 20 AOs and 19 NOs were scanned in a 1.5 T Siemens Magnetom equipped with a standard head coil following previous procedures (Lenroot and Giedd, 2010; Terribilli et al., 2011). The anatomical and 3D T1-weighted images had the following parameters: TR = 7.9, TE = 3.8, ACQ matrix 220 × 220 pixels, voxel size 0.5 × 0.5 × 0.5 mm, 310 sections.

All imaging analysis steps were conducted as described in the VBM pipeline^1^, as follows. The T1-weighted images were normalized to the same stereotaxic space generated from the complete data set using the DARTEL algorithm, which significantly reduces the imprecision of inter-subject recordings. The images were then segmented into white matter, and GM, and non-brain voxels (cerebrospinal fluid). Subsequently, all images were modulated to correct volume changes by Jacobian determinants. Finally, images were smoothed by convolution with an isotropic 8-mm full-width Gaussian kernel at half maximum for statistical analyses.

### Data Analysis

#### Behavioral Data

Demographic and cognitive assessment data were compared between groups through ANOVAs, while categorical variables were assessed *via* chi-square tests.

The mean of correct responses for Task A was compared between groups through a one-way ANOVA.

Contextual indices for Task B were compared using 2 × 2 × 4 repeated-measures ANOVAs, comprising the factors of group (AOs, NOs), condition (congruent, incongruent), and type of emotion (anger, sadness, disgust, fear).

To calculate categorization accuracy, we analyzed the mean of correct responses to determine the emotion conveyed by the face in congruent scenarios (where face and body convey the same emotions) and in incongruent scenarios (where face and body convey different types of emotions). To estimate the contextual index, we analyzed the percentage of times the face was labeled as expressing the contextual body emotion (e.g., when a disgust face in a fear context is labeled as expressing fear), as opposed to any other emotion. We calculated: (a) a measure for congruent context—identical to categorization accuracy for congruent trials; and (b) a measure for incongruent contexts—the degree to which contextual information (i.e., body language) affects facial emotion recognition.

Additionally, and based on the presence of a triple interaction between group, condition, and type of emotion, we ran new independent 2 × 2 ANOVA for each emotion using group (AOs, NOs) as a between-subjects factor and condition (congruent, incongruent) as a within-subjects factor. We followed this procedure to determine, in each emotion, which particular contrast between group and condition could exhibit significant differences. Since the groups differed in educational level and EFs (Table 1), both variables were entered as covariates in the behavioral analyses of tasks A and B, adjusted independently for years of education and IFS scores. Only those effects that remained significant after covariation were reported. Effect sizes were calculated through partial eta squared (*η*^2^).

We ran *post hoc* Tukey analyses to assess significant differences in the contrast of congruency of situations, type of emotions, and group. We used this *post hoc* index considering that the observations to be tested are independent within and among the groups. We assumed that the mean of results should be normally distributed, and that there is equal within-group variance across the groups associated with each mean in the test (homogeneity of variance).

The relationship between EFs, behavioral disturbances, and performance on contextual indices in AOs was explored conducting multiple regression analyses. We ran a regression model for each contextual index. Different measures tracking behavioral disturbances (including months of jail imprisonment and number of relapses) were introduced as predictors in each regression model.

We also assessed whether measures of disorderly conduct in AOs (months of jail sentence and number of relapses) mediated the association between total IFS scores and measures of face-body emotion integration (e.g., categorization accuracy and contextual influence index). To this end, we ran an independent mediation analysis for each face-body emotion integration measure.

#### VBM Data Analysis

To explore regional GM reduction in AOs relative to NOs, we performed a two-sample comparison including total intracranial volume, IFS scores, and years of education as confounding covariates. Additionally, we searched for possible associations between brain morphometry and task performance. To this end, we used the tasks’ performance indexes as regressors and implemented a whole-brain analysis following previous procedures (Santamaría-García et al., 2017; Baez et al., 2018b). The statistical threshold for all whole-brain analyses was set at *p* < 0.001 (extent threshold ≥30 voxels).

##### Relationship Between Brain Morphometry and Contextual Indices

Multiple regression analyses were performed to explore the association between regional GM and measures showing significant between-group differences. To this end, and following previous procedures (Melloni et al., 2016; O’Callaghan et al., 2016; Santamaría-García et al., 2017), we included both AOs and NOs in a single set (all subjects) to increase behavioral variance and statistical power by increasing sample size, giving stability of VBM results, and enhancing the consistency of the anatomical correlates of the cognitive measure analyzed.

In a second stage aimed to assess the specific association between brain volume and behavior, we conducted the same analysis only in AOs. This approach allows exploring which areas are critical for a particular cognitive process (see Melloni et al., 2016; O’Callaghan et al., 2016; Santamaría-García et al., 2017; Shahid et al., 2017). As a complementary analysis, we also examined the specific association between GM volume and behavior in the NO group. For all analyses, total IFS scores, years of education, and total intracranial volume were included as covariates of no-interest (*p* < 0.001 uncorrected, extent threshold ≥30 voxels).

## Results

### Emotion Recognition

#### Task A: Recognition of Context (Body Alone)

**Figure 2:**
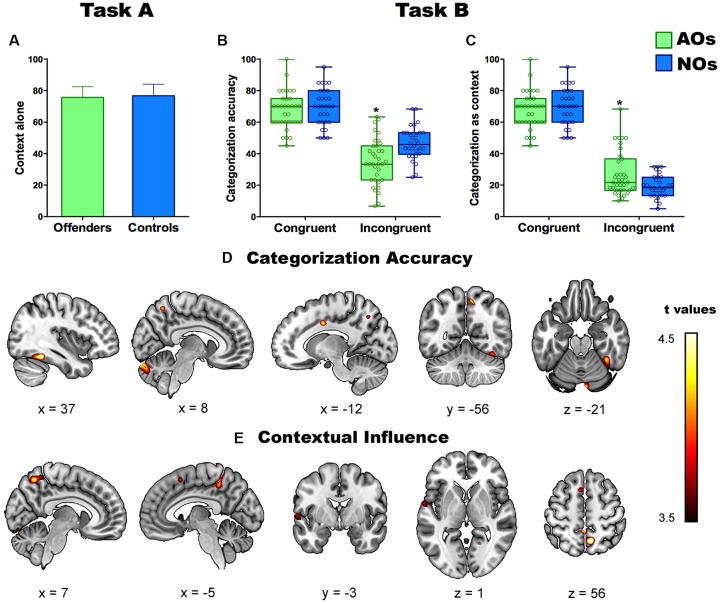
Panel **(A)** shows percentage of correct labeling of emotions in Task A (context alone) in adolescents offenders (AOs) and non-offenders (NOs). Panels **(B,C)** show the performance in the two contextual indices (categorization accuracy and contextual influence) of Task B. Asterisks depict significant differences between groups. Panels **(D,E)** show the significant correlations between gray matter (GM) volume and performance in categorization accuracy **(D)** and contextual influence **(E)** in all subjects. Colored brain areas depict significant brain-behavior correlations at *p* < 0.001 (uncorrected). *Indicates a statistically significant contrast.

This task yielded no type of emotion effect (*F*_(3,189)_ = 1.79, *p* = 0.45, η = 0.01), no group differences (*F*_(1,64)_ = 0.38, *p* = 0.84), and no interaction between emotion and group (*F*_(3,189)_ = 1.25, *p* = 0.49, *η*^2^ = 0.01; Figure 2A). In agreement with a previous study (Kret and de Gelder, 2013), accuracy in each group exceeded 75%.

#### Task B: Contextual Effects

As regards categorization accuracy, ANOVA results revealed a main effect of congruency (*F*_(1,60)_ = 141.91, *p* < 0.0000, *η*^2^ = 0.70), a main effect of type of emotion (*F*_(3,180)_ = 25.79, *p* < 0.000, *η*^2^ = 0.30), an interaction between emotion and group (*F*_(3,180)_ = 4.25, *p* < 0.01, *η*^2^ = 0.06), and a triple interaction among group, congruency, and emotion (*F*_(3.180)_ = 3.45, *p* < 0.01, *η*^2^ = 0.06). A *post hoc* analysis (Tukey’s HSD, MS = 81.24, *df* = 63) showed that AOs performed worse than NOs on incongruent items (*p* < 0.01). No differences were observed for the congruent condition (*p* > 0.3; Figure 2B). The group’s differences in EFs did not explain the aforementioned pattern of results as the contrasts remained significant when it was introduced the EFs as covariate. In particular, it was maintained the main effect of congruency (*F*_(1,60)_ = 11.30, *p* < 0.001, *η*^2^ = 0.16), as well as the main effect of the type of emotion (*F*_(3,180)_ = 5.58, *p* < 0.001, *η*^2^ = 0.08), and the triple interaction among group, congruency and emotion (*F*_(3,180)_ = 3.09, *p* < 0.05, *η*^2^ = 0.05).

Concerning the contextual influence index, ANOVA results revealed a main effect of congruency (*F*_(1,189)_ = 439.15, *p* < 0.0000, *η*^2^ = 0.88), a main effect of type of emotion (*F*_(3,189)_ = 26.6, *p* < 0.000, *η*^2^ = 0.28), an interaction between group and congruency (*F*_(3,189)_ = 11.42, *p* < 0.001, *η*^2^ = 0.15), an interaction between emotion and group (*F*_(3,189)_ = 4.57, *p* < 0.01, *η*^2^ = 0.06), and a triple interaction among group, congruency, and emotion (*F*_(3,189)_ = 4.14, *p* < 0.01, *η*^2^ = 0.06). The *post hoc* analysis (Tukey’s HSD, MS = 85.49, *df* = 63) revealed that, compared to NOs, AOs were significantly more likely to label the facial emotion as that displayed by the context for incongruent items (*p* < 0.01), there being no differences on congruent ones (*p* > 0.4; Figure 2C). As occurred with categorization accuracy, the EFs did not fully explain the pattern of results as most of contrast remained significant after introduced as covariate factor the EFs. In particular, results revealed a significant interaction group by congruency (*F*_(1,62)_ = 5.18, *p* < 0.05, *η*^2^ = 0.07), a main effect of the type of emotion (*F*_(3,180)_ = 2.71, *p* < 0.05, *η*^2^ = 0.04), as well as it was significant the triple interaction among group, congruency and emotion (*F*_(3,180)_ = 2.69, *p* < 0.05, *η*^2^ = 0.03). The rest of contrast did not reach significant results.

A particular pattern of results was observed for both indices (categorization accuracy and contextual influence) for each emotion (anger, fear, disgust, and sadness). In particular, AOs exhibited lower categorization accuracy than NOs in perceiving incongruent situations of negative emotions including sadness and fear. In addition, AOs exhibited a greater contextual influence in the presence of incongruent situations of hostile emotions including disgust and anger.

Five participants of the AO group showed extreme values in the contextual influence index. Significant values in both contextual indices did not change upon removal of those subjects.

##### Contextual Influence Scores Without Outliers

Five participants of the AO group showed extreme values in the contextual influence index as they scored 3 SD above the group’s mean. In general, for both measures (categorization accuracy and contextual influence), the results remained similar to those obtained without excluding the outliers.

In particular, for categorization accuracy, results showed worse performance for AOs than NOs on incongruent items. The analyses showed a main effect of congruency (*F*_(1,58)_ = 3.34, *p* < 0.05, *η*^2^ = 0.07), group (*F*_(1,58)_ = 2.91, *p* < 0.05, *η*^2^ = 0.07) as well as a significant group-by-congruency interaction (*F*_(1,58)_ = 2.45, *p* < 0.05, *η*^2^ = 0.06). A *post hoc* analysis (Tukey’s HSD, MS = 81.24, *df* = 63) showed that AOs performed worse than NOs on incongruent items (*p* < 0.05). No differences in performance were observed for the congruent items (*p* > 0.7).

In line with categorization accuracy outcomes, results of the contextual influence measure showed that AOs performed worse than NOs on incongruent items. The analyses showed a main effect of congruency (*F*_(1,58)_ = 2.15, *p* < 0.05, *η*^2^ = 0.05), but not of group (*F*_(1,58)_ = 1.33, *p* = 0.29, *η*^2^ = 0.01); and a significant group-by-congruency interaction (*F*_(1,58)_ = 2.14, *p* < 0.05, *η*^2^ = 0.06). The *post hoc* analysis (Tukey’s HSD, MS = 85.49, *df* = 63) revealed that the AO group was significantly more able to label the facial emotion as that displayed by the context for incongruent items (*p* < 0.05), there being no differences for the congruent ones (*p* > 0.4).

#### Contextual Indices Analyses for Each Emotion

##### Anger

For categorization accuracy, we observed a main effect of congruency (*F*_(1,63)_ = 5.06, *p* < 0.05, *η*^2^ = 0.76), but not of group (*F*_(1,63)_ = 3.07, *p* = 0.06, *η*^2^ = 0.09). The interaction of group by congruency was also non-significant (*F*_(1,63)_ = 0.47, *p* = 0.64, *η*^2^ = 0.01). For contextual influence, we observed a group-by-congruency interaction (*F*_(1,63)_ = 8.68, *p* < 0.001, *η*^2^ = 0.21), but no effects of congruency (*F*_(1,63)_ = 3.32, *p* = 0.07, *η*^2^ = 0.05) no group (*F*_(1,63)_ = 1.77, *p* = 0.38, *η*^2^ = 0.03). The *post hoc* analysis (Tukey’s HSD, MS = 85.49, *df* = 63) revealed more contextual influence for the AOs group compared to NOs on the incongruent items (*p* < 0.01), without differences for congruent items (*p* > 0.4).

##### Sadness

For categorization accuracy, the only significant result corresponded to the congruency-by-group interaction (*F*_(1,63)_ = 4.81, *p* < 0.01, *η*^2^ = 0.13). No effects of group (*F*_(1,63)_ = 0.9, *p* = 0.41, *η*^2^ = 0.02) or congruency (*F*_(1,63)_ = 0.03, *p* = 0.85, *η*^2^ = 0.01) were observed. The *post hoc* analysis (Tukey’s HSD, MS = 85.49, *df* = 63) revealed worse performance in the AOs group compared to NOs for the incongruent items (*p* < 0.01), without differences for congruent items (*p* > 0.4). Contextual influence analyses showed no significant effects of congruency (*F*_(1,63)_ = 0.02, *p* = 0.96, *η*^2^ = 0.00) or group (*F*_(1,63)_ = 2.08, *p* = 0.13, *η*^2^ = 0.06). The group-by-congruency was also non-significant (*F*_(1,63)_ = 0.21, *p* = 0.81, *η*^2^ = 0.00).

##### Disgust

For categorization accuracy analyses showed no effects of congruency (*F*_(1,63)_ = 1.32, *p* = 0.25, *η*^2^ = 0.02), group (*F*_(1,63)_ = 2.85, *p* = 0.06, *η*^2^ = 0.08), or interactions (*F*_(1,63)_ = 1.94, *p* = 0.15, *η*^2^ = 0.05) were observed. For contextual influence, a main effect of congruency (*F*_(1,63)_ = 5.74, *p* < 0.02, *η*^2^ = 0.08), and an interaction of group × congruency (*F*_(1,63)_ = 4.51, *p* < 0.05, *η*^2^ = 0.12) were observed. No effects of group (*F*_(1,63)_ = 2.83, *p* = 0.09, *η*^2^ = 0.07) were observed. A *post hoc* analysis (Tukey’s HSD, MS = 85.49, *df* = 63) revealed more contextual influence in the AOs group compared to the NOs group for the congruent items (*p* < 0.01), without differences for incongruent items (*p* > 0.42).

##### Fear

For categorization accuracy analyses showed significant effects of congruency (*F*_(1,63)_ = 4.91, *p* < 0.05, *η*^2^ = 0.07), group (*F*_(1,63)_ = 4.26, *p* < 0.05, *η*^2^ = 0.12), and an interaction of group × congruency (*F*_(1,63)_ = 3.6, *p* < 0.05, *η*^2^ = 0.11). A *post hoc* analysis (Tukey’s HSD, MS = 85.49, *df* = 63) revealed worst performance in the AOs group compared to NOs group for the incongruent items (*p* < 0.01), without differences for congruent items (*p* > 0.1). For contextual influence, only a main effect of congruency (*F*_(1,63)_ = 18.5, *p* < 0.001, *η*^2^ = 0.22) was observed. No effects of group (*F*_(1,63)_ = 0.57, *p* = 0.56, *η*^2^ = 0.01) or interaction group × congruency (*F*_(1,63)_ = 0.62, *p* = 0.54, *η*^2^ = 0.01) were observed.

#### Relationship Between Levels of Disorderly Conduct, EFs, and Contextual Indices

A regression model including the total IFS score (as a measure of EFs) and the measures of disorderly conduct (months of jail sentence and number of relapses) reached significant values (*F*_(1,34)_ = 3.23, *p* < 0.05, *R*^2^ = 0.23) and showed that EFs (η = 0.36, *p* < 0.05, *η*^2^ = 0.09), number of relapses (η = −0.23, *p* < 0.05, *η*^2^ = 0.09), and months of jail sentence (η = −0.21, *p* < 0.05, *η*^2^ = 0.09) explained the variance of categorization accuracy index. A similar model over the contextual influence index (*F*_(1,34)_ = 4.21, *p* < 0.05, *R*^2^ = 0.12) showed that its variance was explained by EFs (η = −0.29, *p* < 0.05, *η*^2^ = 0.09) and number of relapses (η = 0.24, *p* < 0.05, *η*^2^ = 0.09), but not months of jail sentence (η = 0.13, *p* = 0.12, *η*^2^ = 0.04).

#### Mediation of Disorderly Conduct in the Association Between EFs and Contextual Indexes

The analyses revealed a significant association between total IFS score and categorization accuracy (*F*_(1,34)_ = 4.58, *p* < 0.05, *R*^2^ = 0.26), which was partially mediated by disorderly conduct measures (relapses and months of jail). In particular, an independent regression using the categorization accuracy as dependent measure, EFs as independent measure, and each disorderly conduct measure as mediation indexes remained significant although the values of *F* decreased in relation to the regression without mediation indexes. On the one hand, the regression using the number of relapses as mediation index showed lower *F* values (*F*_(1,34)_ = 3.23, *p* < 0.05, *R*^2^ = 0.23) but revealed that both EFs (η = 0.27, *p* < 0.05, *η*^2^ = 0.06) and relapses (η = 0.21, *p* < 0.05, *η*^2^ = 0.05) explained the variance of the dependent measure. On the other hand, the regression using months of jail as mediation index showed lower *F* values (*F*_(1,34)_ = 3.12, *p* < 0.05, *R*^2^ = 0.19) and revealed that both EFs (η = 0.36, *p* < 0.01, *η*^2^= 0.04) and months of jail (η = −0.23, *p* < 0.05, *η*^2^= 0.06) explained the variance of categorization accuracy.

Also, a regression using the contextual influence index as dependent measure and EFs as independent measure reached significant values (*F*_(1,34)_ = 5.31, *p* < 0.05, *R*^2^ = 0.38). This association remained significant but with reduced *F* values upon introduction of the relapses as mediation index (*F*_(1,34)_ = 4.21, *p* < 0.05, *R*^2^ = 0.12). This analysis also revealed that both EFs (η = 0.31, *p* < 0.05, *η*^2^ = 0.06) and relapses (η = −0.29, *p* < 0.05, *η*^2^ = 0.06) partially explained the variance of the contextual influence index. The analyses revealed no mediation effect of months of jail on the relationship between EFs and the contextual influence index (*F*_(1,34)_ = 3.88, *p* < 0.05, *R*^2^ = 0.08; i.e., the EFs explained the variance of the contextual influence index (η = 0.21, *p* < 0.02, *η*^2^ = 0.04) but months of jail did not reach significant values (η = −0.11, *p* = 0.21, *η*^2^ = 0.01).

##### Relationship Between EFs and Contextual Indices in AOs and NOs

###### Congruent Items

No significant correlations were observed between categorization accuracy (AOs: *r* = 0.03, *p* = 0.84; NOs: *r* = 0.16, *p* = 0.37) or contextual influence (AOs: *r* = 0.03, *p* = 0.84; NOs: *r* = 0.16, *p* = 0.37) and the total IFS scores in any group.

###### Incongruent Items

Categorization accuracy was significantly associated with IFS scores in both groups (AOs: *r* = 0.40, *p* < 0.01; NOs: *r* = 0.58, *p* < 0.01). Contextual influence indices were significantly associated with total IFS scores in AOs (*r* = −0.38, *p* < 0.05), but not in NOs (*r* = −0.32, *p* = 0.07).

### GM Volume Differences Between AOs and NOs

The comparison between groups (at a threshold of *p* < 0.001 uncorrected) showed that AOs exhibited less GM volume in the postcentral gyrus. Using a more lenient threshold (*p* < 0.01, uncorrected), the comparison showed that AOs also exhibited GM reduction in the bilateral cerebellum, the right precuneus, the angular gyrus, and the precentral gyrus (see Table 3).

**Table 3:** Regions of significant (local maxima) reduced gray matter (GM) volume in AOs compared with NOs.

Region	Cluster *k*	*x*	*y*	*z*	Peak *t*	Peak *z*	*P* Value (uncorrected)
Left postcentral gyrus	1050	−18	−32	80	4.85	4.21	0.00001
Left cerebellum	1108	−32	−93	−33	3.33	3.08	0.0009
Left thalamus	198	−3	−14	20	3.27	3.04	0.001
Right cerebellum	4909	40	−87	−33	3.27	3.03	0.001
Right cerebellum	624	36	−81	44	3.01	2.82	0.002
Right precuneus	235	12	−54	63	2.95	2.77	0.002
Right paracentral lobule	73	10	−22	78	2.67	2.53	0.005

### Relationship Between Brain Morphometry and Contextual Indices (Task B) in AOs and NOs

Regression analyses in AOs and NOs revealed a significant positive association between categorization accuracy and GM volume of the bilateral FG, the left CC, the right precuneus, and the right cerebellum (Figure 2D). A negative correlation also emerged between the contextual influence index and GM volume of the bilateral precuneus, the left STG, and the left SMA (Figure 2E and Table 4).

**Table 4:** Significant brain-behavior associations for both groups together.

Region	Cluster *k*	*x*	*y*	*z*	Peak *t*	Peak *z*	*P* Value (uncorrected)
**Categorization accuracy**							
Right fusiform gyrus	620	38	−52	−20	4.29	3.82	0.00005
Left fusiform gyrus	847	−39	−50	−21	4.41	3.9	0.00004
Right precuneus	129	7	−57	55	4.06	3.66	0.0001
Left cingulate gyrus	98	−14	−3	38	4.07	3.67	0.0001
Right cerebellum	742	8	−82	−21	4.45	3.93	0.00003
**Contextual influence**							
Right precuneus	55	10	−58	56	3.83	3.47	0.0002
Left precuneus	34	0	−42	56	4.16	3.73	0.00008
Left superior temporal gyrus/Rolandic operculum	50	−63	0	4	3.48	3.21	0.0006
Left supplementary motor area	104	−7	13	55	3.46	3.19	0.0006

A similar pattern was observed when analyzing brain-behavior correlations in AOs only. In particular, the analyses showed a significant positive association between categorization accuracy and GM volume of the bilateral FG. In addition, a negative correlation also emerged between the contextual influence index and GM volume of the bilateral precuneus and CC (see Table 5).

**Table 5:** Significant brain-behavior associations in AOs.

Region	Cluster *k*	*x*	*y*	*z*	Peak *t*	Peak *z*	*P* Value (uncorrected)
**Categorization accuracy**							
Right fusiform gyrus	109	45	−46	−9	5.64	4.07	0.00001
Left fusiform gyrus	60	−38	−48	−20	4.38	3.46	0.0001
**Contextual influence**							
Precuneus/mid cingulate cortex	107	0	42	62	5.07	3.81	0.00003
Left precuneus	101	−2	−42	60	4.65	3.64	0.00008
Right precuneus	102	2	−50	66	3.85	3.19	0.0005

For NOs, regression analyses revealed a significant positive association between categorization accuracy and GM volume of the right FG. A negative correlation also emerged between the contextual influence index and GM volume of right superior temporal lobe and right insula (Table 6).

**Table 6:** Significant brain-behavior associations in NOs.

Region	Cluster *k*	*x*	*y*	*z*	Peak *t*	Peak *z*	*P* Value (uncorrected)
**Categorization accuracy**							
Right fusiform gyrus	32	38	−64	−9	5.12	3.89	0.00003
**Contextual influence**							
Right superior temporal gyrus	151	31	−55	3	4.15	3.41	0.0003
Right insula	55	28	−22	10	4.08	3.38	0.0003
Right precuneus	102	2	−50	66	3.85	3.19	0.0005

## Discussion

This study examined how contextual body language modulates facial emotion recognition in AOs, also exploring neuroanatomical markers of such integration. Results showed a greater influence of context (body language) on face emotion recognition in AOs compared to NOs, mainly affecting the integration of incongruent trials. This increased context-sensitivity was associated to measures of EFs and disruptive behaviors, as well as to the morphology of brain areas involved in body perception, contextual integration, and processing of conflicting emotional stimuli.

In agreement with previous research on similar populations (Kret and de Gelder, 2013), results of Task A showed no between-group differences in recognizing emotional cues conveyed by bodies. Instead, Task B revealed divergent profiles between AOs and NOs for both contextual measures. While the two groups performed similarly on congruent face-body trials, they differed when responding to incongruent contexts. Particularly, AOs exhibited poorer emotion categorization accuracy than NOs and higher susceptibility to the contextual influence of body cues on emotion identification. Although the mismatch between bodily and facial information affected emotion recognition in both groups, AOs were more prone to labeling faces in terms of the emotion conveyed by body language.

Analyses of face-body integration for each type of emotions reveal two major differences between groups in both contextual indices. First, AOs exhibited poor categorization accuracy for labeling sadness and fear emotions in incongruent situations compared to NOs group. No differences between groups were observed in contextual influence measure for those emotions. Second, AOs had more contextual influence than the NOs group for the incongruent items of hostile emotions including anger and disgust. Categorization accuracy analyses of these emotions did not reveal significant group differences.

The current results seem to suggest that both types of alterations might be coexist in AOs, as they presented reduced categorization accuracy of perceiving negative emotions alongside a greater influence of body context for hostile emotions (disgust and anger). Together, those results reveal major difficulties in processing emotions in incongruent context, as AOs tend to be more affected by body signals.

Our results confirm the increased sensitivity of ecological tasks tapping contextual information (Baez et al., 2012, 2017a; Ibañez and Manes, 2012; Amoruso et al., 2013). Moreover, they suggest that, relative to NOs, AOs tend to overvalue body signals in contextual emotion recognition. These findings align with previous studies on adult offenders (Grèzes et al., 2007) showing that, in incongruent scenarios, the judgment of target emotions may be hampered and become biased toward body-based and contextual emotional cues. Furthermore, our results are consistent with a previous study reporting impaired contextual emotional integration in AOs *via* emotional situations staged in real-life videos (Gonzalez-Gadea et al., 2014).

The pattern of results in each emotion supports previous theories on the relationship between emotion recognition and aggressive behaviors in offenders (Sato et al., 2009; Bowen et al., 2014). Those theories postulate that offenders present defensive and maladaptive behaviors associated to both greater sensitivity towards negative signals, such as disgust or anger emotions (Dodge et al., 1990), and reduced ability in recognizing social signals of distress—including negative emotions such as sadness and fear (Blair et al., 2001; Bowen et al., 2014). These patterns have also been associated with reduced activity of frontal networks (Best et al., 2002; Sato et al., 2009).

Our results align with previous studies showing that implicit integration of emotional signals transcends facial information and relies on the dynamic perception of internal and external stimuli, including interoceptive signals, complex sensory-perceptual inputs, and contextual cues (de Gelder et al., 1999, 2004; Aviezer et al., 2012; Van den Stock et al., 2014; Wieser and Keil, 2014; Ferrari et al., 2016; Davies-Thompson et al., 2018). Furthermore, in agreement with previous studies, our results support the idea that body signals are highly salient in the process of perceiving relevant emotional information from context (Aviezer et al., 2009, 2012; Bachmann et al., 2018). This is particularly relevant in offenders, considering that those subjects are continuously tracking potential threats in the environment.

Previous studies associated facial emotion recognition in AOs to disruptive behaviors (Fairchild et al., 2009, 2010; Sato et al., 2009). As predicted, our results showed that, in these subjects, the latter variable (i.e., number relapses and months of jail sentence) was related to an over-estimation of body signals. Misinterpretation of body cues may impel AOs to activate a “fight mode,” increasing behavioral reactivity and externalized behaviors. This explanation is supported by evidence that adult offenders tend to infer hostile messages in undifferentiated body cues (Kret and de Gelder, 2013). Thus, externalizing behaviors and poor appraisal of contextual information could be considered a key determinant of AOs’ social behavior.

Also, in agreement with previous studies (Escobar et al., 2014; Ibáñez et al., 2014), we showed that EFs are associated with emotion recognition. However, the described pattern of results for both contextual measures remained significant after covariation with EFs and education level, suggesting that contextual integration effects in AOs are not completely explained by (domain-general) cognitive difficulties.

Furthermore, mediation analyses revealed that disorderly conduct mediated the relationship between EFs and contextual integration tasks. The presence of conduct disorders could affect emotion perception by introducing less adaptive bodily and behavioral postures for perceiving emotional information. In fact, previous studies have suggested that emotional information is situated and embodied, the perception of emotional cues is supported by congruent corporal dynamic (Neal and Chartrand, 2011). Thus, the presence of an altered behavioral and corporal disposition, as occurs in AOs with disorderly conduct, could affect more flexible and embodied forms of emotional perception.

Additionally, our results point to a dynamic cycle of cognitive-behavioral functioning in AOs. These subjects usually exhibit alterations in executive functioning, emotional regulation, and emotional perception, as well as a tendency to overvalue body emotional signals and detect them as threats. Those dysfunctions are implicitly associated to externalizing behaviors including conduct disorders (Tamietto and de Gelder, 2010). Arguably, AOs with more severe behavior disorders could exhibit greater reactivity in perceiving the most salient behavioral and emotional signals (in this case, body signals over other subtle signals), as they can convey implicit threats. Future studies should assess how AOs perceive and integrate subtle emotional information and non-threatening signals in comparison to healthy individuals. Furthermore, the extent to which conduct disturbances affect emotional integration processes should be more actively explored in future studies.

Arguably, subjects with more externalizing and anxiety symptoms tend to recognize ambiguous signals as threatening cues (Kret and de Gelder, 2013; Philipp-Wiegmann et al., 2017). Thus, negative emotions, rather than positive ones, seem to be more sensitive in revealing difficulties for integrating contextual cues. Given that this study only included negative emotions, future studies in AOs should assess contextual effects on positive emotion recognition, and relevant structural correlates.

Crucially, our results revealed brain volume differences between AOs and NOs. In agreement with previously reported evidence (Rogers and De Brito, 2016; Budhiraja et al., 2017), the AOs group showed reduced GM volumes in the right precuneus, the angular gyrus, and the precentral gyrus. These areas have been previously implicated in different socio-cognitive operations, including the detection of social and emotional signals, mentalizing processes, regulation of social behaviors, and moral judgment (Cope et al., 2014).

Concerning to the association of contextual indexes and GM, the higher the categorization accuracy was associated to a higher the GM volume in the bilateral FG, the left CC, the right cerebellum, and the right precuneus. The FG is classically implicated in face (Kanwisher and Yovel, 2006) and body (Schwarzlose et al., 2005) perception. Crucially, this area is sensitive to emotional information conveyed by both types of stimulus (Peelen et al., 2007). Compatibly, our findings suggest that, beyond its putative functional roles, FG anatomy is related to face and body recognition skills (Onitsuka et al., 2003). Also, the association between cerebellar GM volume and categorization accuracy confirms the involvement of this area in categorical perception of faces and body (Van den Stock et al., 2014), and its role in motor resonance when body postures revealing movement are perceived (Sokolov et al., 2010). Furthermore, the relationship with CC also supports the role of this area in processing conflictive emotional information (Etkin et al., 2011), mobilizing defensive responses in the presence of conflictive emotions (Pereira et al., 2010), and integrating contextualized emotional information (Maren et al., 2013). Finally, the association with GM volume in the precuneus may reflect this region’s involvement in modulating attention towards dominant facial cues during emotion perception (Zhang and Li, 2012).

Furthermore, we found that a greater bias towards body information (contextual influence) was associated to lower GM volume of the left STG and SMA, and the bilateral precuneus. Consistent with our results, the STG has been implicated in processing affective features of faces and bodies (van de Riet et al., 2009). For their part, the SMA and the precuneus are involved in motor resonance of complex emotional stimuli (Orgs et al., 2016) and attention to emotional information in context (Van den Stock et al., 2011). Of note, in our study, the GM volume of precuneus was associated to both contextual measures. This may suggest a crucial role of such a region in tasks requiring high attentional efforts for integrating conflictive emotional stimuli conveyed by faces and bodies.

For NOs, in line with previous studies (Van den Stock et al., 2014; Kumfor et al., 2018), our results revealed an association between categorization accuracy and GM volume of the right FG. Furthermore, confirming previous evidence, our study revealed an association between the contextual influence index and GM volume of the STG (Kumfor et al., 2018), a region involved in processing of social and emotional signals. Our results also revealed a significant association between the contextual influence index and GM volume in the precuneus and the insula, two areas involved in the processing of emotional body language (Ahmed et al., 2015) and the monitoring and representation of bodily states (Karnath et al., 2005; Peelen and Downing, 2007; Peelen et al., 2007), respectively.

Summarizing, brain-behavior correlations showed that the integration of body cues in emotion recognition are supported by brain areas involved in body-face recognition (FG; Peelen et al., 2007), emotion processing (CC, STG, FG; Pereira et al., 2010), contextual integration (precuneus, STG; van de Riet et al., 2009), and motor resonance (cerebellum, SMA; de Gelder, 2006). This pattern of associations for both measures was maintained when AOs were analyzed separately. These results indicate that the integration of bodily and facial emotional cues in incongruent scenarios is a complex process recruiting areas implicated in tracking conflicting emotion information, beyond classical areas subserving the recognition of bodily and facial signals.

One limitation of our study consists in the lack of standardized measures tracking disruptive and externalized behaviors, which could help to clarify the association between face-body emotion recognition and behavioral disorders in AOs. Future studies might explore the interplay between contextual emotional recognition and levels of disorderly conduct in this population through standardized assessment of relevant factors.

Another potential limitation of our study is that the groups differed in terms of EF scores and educational level. Concerning EFs, previous studies have reported reduced scores in executive tasks in AOs compared to healthy controls (Gonzalez-Gadea et al., 2014; Seruca and Silva, 2016). Additionally, note that AOs typically present high school dropout rates and poor academic achievements (Breslau et al., 2009; McLeod et al., 2012). However, as shown by the covariation and regression analyses, EFs and educational differences did not explain the group’s differences in the categorization accuracy and contextual influence measures. Future studies should explore the extent to which integration of emotional information from faces and bodies modulate particular subtypes of EFs, including emotion regulation and cognitive control. In fact, previous studies have revealed differences in cognitive control flexibility and planning in AOs with different type of offenses (Seruca and Silva, 2016).

Although we targeted a particular sample of Colombian AOs, our sample shared demographic factors, and the profile of offenses to samples assessed in previous studies (Brazil et al., 2013; Zou et al., 2013; Vilà-Balló et al., 2015), including reports assessing specific cognitive, emotional, and socio- cognitive processes (see Gonzalez-Gadea et al., 2014; Baez et al., 2018a). In fact, the AOs tested herein resembled those of previous samples from other countries in terms of demography (sharing sex, ages, socio-cultural background), behavioral disturbances (including months of jail and relapses), and type of offenses (including offenses such as homicide, sexual related offences, extortion among other offenses; see Brazil et al., 2013; Zou et al., 2013; Vilà-Balló et al., 2015). Nevertheless, the assessment of socio-cognitive processes in AOs should include a more varied sample of offenders from different sociocultural backgrounds. Thus, future research should assess to what extent the particular contextual and social conditions experienced by AOs in this study modulate the processes under study.

To summarize in this study, we assessed body-face and contextual emotional integration in AOs and NOs. Our results suggest that AOs tend to overvalue body signals in contextual emotion recognition. The sensitivity to body information in this group was associated to GM volume of brain areas involved in integrating bodily cues, updating contextual information, and processing emotion-laden stimuli in conflictive contexts. Finally, our results pave the way for a better understanding of the association between contextual emotion recognition, behavioral regulation and externalized behaviors in AOs. Further research along these lines may provide insights on potential avenues for rehabilitation of social cognition difficulties in AOs (e.g., by providing complementary contextual information to enhance understanding of social cues).

## Data Availability

The dataset generated by this study is available on request to the corresponding author.

## Author Contributions

HS-G, SB, AI, CI, MP, SM, and AG developed the study concept and the study design. MP, SM, and CA performed testing and data collection. SB, HS-G, and MP-S performed the data analysis and interpretation under the supervision of SB. HS-G, AG, and SB drafted the manuscript. AI and AG provided critical revisions. All authors have participated sufficiently in the work and approved the final version of the manuscript for submission.

## Conflict of Interest Statement

The authors declare that the research was conducted in the absence of any commercial or financial relationships that could be construed as a potential conflict of interest.

## Footnotes

^1^https://www.fil.ion.ucl.ac.uk/~john/misc/VBMclass10.pdf

## References

[B1] Adolfi F. Couto B. Richter F. Decety J. Lopez J. Sigman M.. (2016). Convergence of interoception, emotion, and social cognition: a twofold fMRI meta-analysis and lesion approach. Cortex 88, 124–142. 10.1016/j.cortex.2016.12.019 28088652

[B2] Ahmed S. P. Bittencourt-Hewitt A. Sebastian C. L. (2015). Neurocognitive bases of emotion regulation development in adolescence. Dev. Cogn. Neurosci. 15, 11–25. 10.1016/j.dcn.2015.07.006 26340451 PMC6989808

[B3] Amoruso L. Gelormini C. Aboitiz F. Alvarez Gonzalez M. Manes F. Cardona J. F.. (2013). N400 ERPs for actions: building meaning in context. Front. Hum. Neurosci. 7:57. 10.3389/fnhum.2013.00057 23459873 PMC3586681

[B4] Ashburner J. Friston K. J. (2000). Voxel-based morphometry—the methods. Neuroimage 11, 805–821. 10.1006/nimg.2000.0582 10860804

[B5] Aviezer H. Bentin S. Hassin R. R. Meschino W. S. Kennedy J. Grewal S.. (2009). Not on the face alone: perception of contextualized face expressions in Huntington’s disease. Brain 132, 1633–1644. 10.1093/brain/awp067 19451178 PMC2724912

[B6] Aviezer H. Hassin R. R. Ryan J. Grady C. Susskind J. Anderson A.. (2008). Angry, disgusted, or Afraid? Studies on the malleability of emotion perception. Psychol. Sci. 19, 724–732. 10.1111/j.1467-9280.2008.02148.x 18727789

[B7] Aviezer H. Trope Y. Todorov A. (2012). Body cues, not facial expressions, discriminate between intense positive and negative emotions. Science 338, 1225–1229. 10.1126/science.1224313 23197536

[B8] Bachmann J. Munzert J. Krüger B. (2018). Neural underpinnings of the perception of emotional states derived from biological human motion: a review of neuroimaging research. Front. Psychol. 9:1763. 10.3389/fpsyg.2018.01763 30298036 PMC6160569

[B10] Baez S. García A. M. Ibanez A. (2017a). The social context network model in psychiatric and neurological diseases. Curr. Top. Behav. Neurosci. 30, 379–396. 10.1007/7854_2016_443 27130326

[B12] Baez S. Herrera E. García A. Manes F. Young L. Ibáñez A. (2017b). Outcome-oriented moral evaluation in terrorists. Nat. Hum. Behav. 1:0165 10.1038/s41562-017-0165

[B11] Baez S. Herrera E. García A. M. Huepe D. Santamaría-García H. Ibáñez A. (2018a). Increased moral condemnation of accidental harm in institutionalized adolescents. Sci. Rep. 8:11609. 10.1038/s41598-018-29956-9 30072749 PMC6072742

[B14] Baez S. Pino M. Berrío M. Santamaría-García H. Sedeño L. García A. M.. (2018b). Corticostriatal signatures of schadenfreude: evidence from Huntington’s disease. J. Neurol. Neurosurg. Psychiatry 89, 112–116. 10.1136/jnnp-2017-316055 28765320

[B9] Baez S. Ibanez A. (2014). The effects of context processing on social cognition impairments in adults with Asperger’s syndrome. Front. Neurosci. 8:270. 10.3389/fnins.2014.00270 25232301 PMC4153041

[B13] Baez S. Ibanez A. Gleichgerrcht E. Perez A. Roca M. Manes F.. (2014). The utility of IFS (INECO Frontal Screening) for the detection of executive dysfunction in adults with bipolar disorder and ADHD. Psychiatry Res. 216, 269–276. 10.1016/j.psychres.2014.01.020 24582774

[B15] Baez S. Rattazzi A. Gonzalez-Gadea M. L. Torralva T. Vigliecca N. S. Decety J.. (2012). Integrating intention and context: assessing social cognition in adults with Asperger syndrome. Front. Hum. Neurosci. 6:302. 10.3389/fnhum.2012.00302 23162450 PMC3492863

[B16] Barrett L. F. Mesquita B. Gendron M. (2011). Context in emotion perception. Curr. Dir. Psychol. Sci. 20, 286–290. 10.1177/0963721411422522

[B17] Best M. Williams J. M. Coccaro E. F. (2002). Evidence for a dysfunctional prefrontal circuit in patients with an impulsive aggressive disorder. Proc. Natl. Acad. Sci. U S A 99, 8448–8453. 10.1073/pnas.112604099 12034876 PMC123087

[B18] Blair R. J. Colledge E. Murray L. Mitchell D. G. (2001). A selective impairment in the processing of sad and fearful expressions in children with psychopathic tendencies. J. Abnorm. Child Psychol. 29, 491–498. 10.1023/A:1012225108281 11761283

[B19] Bowen K. L. Morgan J. E. Moore S. C. van Goozen S. H. M. (2014). Young offenders’ emotion recognition dysfunction across emotion intensities: explaining variation using psychopathic traits, conduct disorder and offense severity. J. Psychopathol. Behav. Assess. 36, 60–73. 10.1007/s10862-013-9368-z 24610972 PMC3935119

[B20] Brazil I. A. Maes J. H. Scheper I. Bulten B. H. Kessels R. P. Verkes R. J.. (2013). Reversal deficits in individuals with psychopathy in explicit but not implicit learning conditions. J. Psychiatry Neurosci. 38, E13–E20. 10.1503/jpn.120152 23552501 PMC3692728

[B21] Breslau J. Miller E. Breslau N. Bohnert K. Lucia V. Schweitzer J. (2009). The impact of early behavior disturbances on academic achievement in high school. Pediatrics 123, 1472–1476. 10.1542/peds.2008-1406 19482756 PMC2778327

[B22] Budhiraja M. Savic I. Lindner P. Jokinen J. Tiihonen J. Hodgins S. (2017). Brain structure abnormalities in young women who presented conduct disorder in childhood/adolescence. Cogn. Affect. Behav. Neurosci. 17, 869–885. 10.3758/s13415-017-0519-7 28695488 PMC5548815

[B23] Burnett S. Sebastian C. Cohen Kadosh K. Blakemore S.-J. (2011). The social brain in adolescence: evidence from functional magnetic resonance imaging and behavioural studies. Neurosci. Biobehav. Rev. 35, 1654–1664. 10.1016/j.neubiorev.2010.10.011 21036192 PMC4538788

[B24] Burra N. Hervais-Adelman A. Celeghin A. de Gelder B. Pegna A. J. (2017). Affective blindsight relies on low spatial frequencies. Neuropsychologia [Epub ahead of print]. 10.1016/j.neuropsychologia.2017.10.009 28993236

[B25] Cope L. M. Ermer E. Nyalakanti P. K. Calhoun V. D. Kiehl K. A. (2014). Paralimbic gray matter reductions in incarcerated adolescent females with psychopathic traits. J. Abnorm. Child Psychol. 42, 659–668. 10.1007/s10802-013-9810-4 24682609 PMC3976761

[B26] Couto B. Adolfi F. Velasquez M. Mesow M. Feinstein J. Canales-Johnson A.. (2015). Heart evoked potential triggers brain responses to natural affective scenes: a preliminary study. Auton. Neurosci. 193, 132–137. 10.1016/j.autneu.2015.06.006 26188392

[B27] Davies-Thompson J. Elli G. V. Rezk M. Benetti S. van Ackeren M. Collignon O. (2018). Hierarchical brain network for face and voice integration of emotion expression. Cereb. Cortex [Epub ahead of print]. 10.1093/cercor/bhy240 30272134

[B28] de Gelder B. (2006). Towards the neurobiology of emotional body language. Nat. Rev. Neurosci. 7, 242–249. 10.1038/nrn1872 16495945

[B29] de Gelder B. Bocker K. B. Tuomainen J. Hensen M. Vroomen J. (1999). The combined perception of emotion from voice and face: early interaction revealed by human electric brain responses. Neurosci. Lett. 260, 133–136. 10.1016/s0304-3940(98)00963-x 10025717

[B30] de Gelder B. Meeren H. K. Righart R. van den Stock J. van de Riet W. A. Tamietto M. (2006). Beyond the face: exploring rapid influences of context on face processing. Prog. Brain Res. 155, 37–48. 10.1016/S0079-6123(06)55003-4 17027378

[B31] de Gelder B. Snyder J. Greve D. Gerard G. Hadjikhani N. (2004). Fear fosters flight: a mechanism for fear contagion when perceiving emotion expressed by a whole body. Proc. Natl. Acad. Sci. U S A 101, 16701–16706. 10.1073/pnas.0407042101 15546983 PMC528902

[B66] de Pedraza F. G. de Rincón D. M. Montealegre G. (2012). Validación de la prueba de J. C. Raven: matrices progresivas y de la prueba ACE para estudiantes de primer año universitario. Rev. Colomb. Psicol. 5, 129–136. 10.15446/rcp

[B32] Diano M. Celeghin A. Bagnis A. Tamietto M. (2017). Amygdala response to emotional stimuli without awareness: facts and interpretations. Front. Psychol. 7:2029. 10.3389/fpsyg.2016.02029 28119645 PMC5222876

[B33] Dodge K. A. Price J. M. Bachorowski J. A. Newman J. P. (1990). Hostile attributional biases in severely aggressive adolescents. J. Abnorm. Psychol. 99, 385–392. 10.1037//0021-843x.99.4.385 2266213

[B34] Escobar M. J. Huepe D. Decety J. Sedeño L. Messow M. K. Baez S.. (2014). Brain signatures of moral sensitivity in adolescents with early social deprivation. Sci. Rep. 4:5354. 10.1038/srep05354 24942045 PMC5381535

[B35] Etkin A. Egner T. Kalisch R. (2011). Emotional processing in anterior cingulate and medial prefrontal cortex. Trends Cogn. Sci. 15, 85–93. 10.1016/j.tics.2010.11.004 21167765 PMC3035157

[B36] Fairchild G. Stobbe Y. van Goozen S. H. Calder A. J. Goodyer I. M. (2010). Facial expression recognition, fear conditioning, and startle modulation in female subjects with conduct disorder. Biol. Psychiatry 68, 272–279. 10.1016/j.biopsych.2010.02.019 20447616 PMC2954286

[B37] Fairchild G. Van Goozen S. H. Calder A. J. Stollery S. J. Goodyer I. M. (2009). Deficits in facial expression recognition in male adolescents with early-onset or adolescence-onset conduct disorder. J. Child Psychol. Psychiatry 50, 627–636. 10.1111/j.1469-7610.2008.02020.x 19432683 PMC2737612

[B38] Fairchild G. Van Goozen S. H. Stollery S. J. Goodyer I. M. (2008). Fear conditioning and affective modulation of the startle reflex in male adolescents with early-onset or adolescence-onset conduct disorder and healthy control subjects. Biol. Psychiatry 63, 279–285. 10.1016/j.biopsych.2007.06.019 17765205

[B39] Ferrari C. Lega C. Vernice M. Tamietto M. Mende-Siedlecki P. Vecchi T.. (2016). The dorsomedial prefrontal cortex plays a causal role in integrating social impressions from faces and verbal descriptions. Cereb. Cortex 26, 156–165. 10.1093/cercor/bhu186 25165063

[B40] Frith C. (2009). Role of facial expressions in social interactions. Philos. Trans. R. Soc. Lond. B Biol. Sci. 364, 3453–3458. 10.1098/rstb.2009.0142 19884140 PMC2781887

[B42] Gonzalez-Gadea M. L. Herrera E. Parra M. Gomez Mendez P. Baez S. Manes F.. (2014). Emotion recognition and cognitive empathy deficits in adolescent offenders revealed by context-sensitive tasks. Front. Hum. Neurosci. 8:850. 10.3389/fnhum.2014.00850 25374529 PMC4204464

[B43] Grèzes J. Pichon S. de Gelder B. (2007). Perceiving fear in dynamic body expressions. Neuroimage 35, 959–967. 10.1016/j.neuroimage.2006.11.030 17270466

[B44] Hassin R. R. Aviezer H. Bentin S. (2013). Inherently ambiguous: facial expressions of emotions, in context. Emot. Rev. 5, 60–65. 10.1177/1754073912451331

[B45] Hubble K. Bowen K. L. Moore S. C. van Goozen S. H. (2015). Improving negative emotion recognition in young offenders reduces subsequent crime. PLoS One 10:e0132035. 10.1371/journal.pone.0132035 26121148 PMC4486167

[B47] Ibáñez A. Aguado J. Baez S. Huepe D. Lopez V. Ortega R.. (2014). From neural signatures of emotional modulation to social cognition: individual differences in healthy volunteers and psychiatric participants. Soc. Cogn. Affect. Neurosci. 9, 939–950. 10.1093/scan/nst067 23685775 PMC4090956

[B46] Ibañez A. Manes F. (2012). Contextual social cognition and the behavioral variant of frontotemporal dementia. Neurology 78, 1354–1362. 10.1212/wnl.0b013e3182518375 22529204 PMC3335455

[B48] Jusyte A. Schonenberg M. (2017). Impaired social cognition in violent offenders: perceptual deficit or cognitive bias? Eur. Arch. Psychiatry Clin. Neurosci. 267, 257–266. 10.1007/s00406-016-0727-0 27623869

[B49] Kanwisher N. Yovel G. (2006). The fusiform face area: a cortical region specialized for the perception of faces. Philos. Trans. R. Soc. Lond. B Biol. Sci. 361, 2109–2128. 10.1098/rstb.2006.1934 17118927 PMC1857737

[B50] Karnath H. O. Baier B. Nägele T. (2005). Awareness of the functioning of one’s own limbs mediated by the insular cortex? J. Neurosci. 25, 7134–7138. 10.1523/JNEUROSCI.1590-05.2005 16079395 PMC6725240

[B51] Kret M. E. de Gelder B. (2013). When a smile becomes a fist: the perception of facial and bodily expressions of emotion in violent offenders. Exp. Brain Res. 228, 399–410. 10.1007/s00221-013-3557-6 23828232 PMC3710410

[B52] Kuin N. C. Masthoff E. D. M. Munafo M. R. Penton-Voak I. S. (2017). Perceiving the evil eye: investigating hostile interpretation of ambiguous facial emotional expression in violent and non-violent offenders. PLoS One 12:e0187080. 10.1371/journal.pone.0187080 29190802 PMC5708671

[B53] Kumfor F. Ibañez A. Hutchings R. Hazelton J. Hodges J. Piguet O. (2018). Beyond the face: how context modulates emotion processing in frontotemporal dementia subtypes. Brain 141, 1172–1185. 10.1093/brain/awy002 29394332

[B54] Lenroot R. K. Giedd J. N. (2010). Sex differences in the adolescent brain. Brain Cogn. 72, 46–55. 10.1016/j.bandc.2009.10.008 19913969 PMC2818549

[B55] Maier M. E. di Pellegrino G. (2012). Impaired conflict adaptation in an emotional task context following rostral anterior cingulate cortex lesions in humans. J. Cogn. Neurosci. 24, 2070–2079. 10.1162/jocn_a_00266 22721382

[B56] Maren S. Phan K. L. Liberzon I. (2013). The contextual brain: implications for fear conditioning, extinction and psychopathology. Nat. Rev. Neurosci. 14, 417–428. 10.1038/nrn3492 23635870 PMC5072129

[B57] McLeod J. D. Uemura R. Rohrman S. (2012). Adolescent mental health, behavior problems, and academic achievement. J. Health Soc. Behav. 53, 482–497. 10.1177/0022146512462888 23197485 PMC3752654

[B58] Melloni M. Billeke P. Baez S. Hesse E. de la Fuente L. Forno G.. (2016). Your perspective and my benefit: multiple lesion models of self-other integration strategies during social bargaining. Brain 139, 3022–3040. 10.1093/brain/aww231 27679483

[B59] Neal D. T. Chartrand T. L. (2011). Embodied emotion perception: amplifying and dampening facial feedback modulates emotion perception accuracy. Soc. Psychol. Pers. Sci. 2, 673–678. 10.1177/1948550611406138

[B60] Nunes D. Monteiro L. Lopes E. (2014). INECO frontal screening: a tool to assess executive functions in depression. Psicol. Clin. 26, 177–196. 10.1590/S0103-56652014000200011

[B61] O’Callaghan C. Bertoux M. Irish M. Shine J. M. Wong S. Spiliopoulos L.. (2016). Fair play: social norm compliance failures in behavioural variant frontotemporal dementia. Brain 139, 204–216. 10.1093/brain/awv315 26503957

[B62] Onitsuka T. Shenton M. E. Kasai K. Nestor P. G. Toner S. K. Kikinis R.. (2003). Fusiform gyrus volume reduction and facial recognition in chronic schizophrenia. Arch. Gen. Psychiatry 60, 349–355. 10.1001/archpsyc.60.4.349 12695311

[B63] Oosterhof N. N. Todorov A. (2008). The functional basis of face evaluation. Proc. Natl. Acad. Sci. U S A 105, 11087–11092. 10.1073/pnas.0805664105 18685089 PMC2516255

[B64] Orgs G. Dovern A. Hagura N. Haggard P. Fink G. R. Weiss P. H. (2016). Constructing visual perception of body movement with the motor cortex. Cereb. Cortex 26, 440–449. 10.1093/cercor/bhv262 26534907 PMC4677987

[B65] Passamonti L. Fairchild G. Goodyer I. M. Hurford G. Hagan C. C. Rowe J. B.. (2010). Neural abnormalities in early-onset and adolescence-onset conduct disorder. Arch. Gen. Psychiatry 67, 729–738. 10.1001/archgenpsychiatry.2010.75 20603454 PMC4471104

[B68] Peelen M. V. Atkinson A. P. Andersson F. Vuilleumier P. (2007). Emotional modulation of body-selective visual areas. Soc. Cogn. Affect. Neurosci. 2, 274–283. 10.1093/scan/nsm023 18985133 PMC2566760

[B67] Peelen M. V. Downing P. E. (2007). The neural basis of visual body perception. Nat. Rev. Neurosci. 8, 636–648. 10.1038/nrn2195 17643089

[B69] Pereira M. de Oliveira L. Erthal F. Joffily M. Mocaiber I. Volchan E.. (2010). Emotion affects action: midcingulate cortex as a pivotal node of interaction between negative emotion and motor signals. Cogn. Affect. Behav. Neurosci. 10, 94–106. 10.3758/cabn.10.1.94 20233958 PMC2875262

[B70] Philipp-Wiegmann F. Rösler M. Retz-Junginger P. Retz W. (2017). Emotional facial recognition in proactive and reactive violent offenders. Eur. Arch. Psychiatry Clin. Neurosci. 267, 687–695. 10.1007/s00406-017-0776-z 28258396

[B71] Piotrowska P. J. Stride C. B. Croft S. E. Rowe R. (2015). Socioeconomic status and antisocial behaviour among children and adolescents: a systematic review and meta-analysis. Clin. Psychol. Rev. 35, 47–55. 10.1016/j.cpr.2014.11.003 25483561

[B72] Piquero A. R. Jennings W. G. Diamond B. Reingle J. M. (2015). A systematic review of age, sex, ethnicity, and race as predictors of violent recidivism. Int. J. Offender Ther. Comp. Criminol. 59, 5–26. 10.1177/0306624x13514733 24335783

[B73] Poyo Solanas M. Zhan M. Vaessen M. Hortensius R. Engelen T. de Gelder B. (2018). Looking at the face and seeing the whole body. Neural basis of combined face and body expressions. Soc. Cogn. Affect. Neurosci. 13, 135–144. 10.1093/scan/nsx130 29092076 PMC5793719

[B74] Raven J. C. (1960). Guide to Standard Progressive Matrices. London: HK Lewis.

[B75] Rogers J. C. De Brito S. A. (2016). Cortical and subcortical gray matter volume in youths with conduct problems: a meta-analysis. JAMA Psychiatry 73, 64–72. 10.1001/jamapsychiatry.2015.2423 26650724

[B76] Santamaría-García H. Baez S. Reyes P. Santamaría-García J. A. Santacruz-Escudero J. M. Matallana D.. (2017). A lesion model of envy and Schadenfreude: legal, deservingness and moral dimensions as revealed by neurodegeneration. Brain 140, 3357–3377. 10.1093/brain/awx269 29112719 PMC5841144

[B77] Sato W. Uono S. Matsuura N. Toichi M. (2009). Misrecognition of facial expressions in delinquents. Child Adolesc. Psychiatry Ment. Health 3:27. 10.1186/1753-2000-3-27 19765274 PMC2756248

[B78] Schwarzlose R. F. Baker C. I. Kanwisher N. (2005). Separate face and body selectivity on the fusiform gyrus. J. Neurosci. 25, 11055–11059. 10.1523/JNEUROSCI.2621-05.2005 16306418 PMC6725864

[B79] Seruca T. Silva C. F. (2016). Executive functioning in criminal behavior: differentiating between types of crime and exploring the relation between shifting, inhibition, and anger. Int. J. Forensic Ment. Health 15, 235–246. 10.1080/14999013.2016.1158755

[B80] Shahid H. Sebastian R. Schnur T. T. Hanayik T. Wright A. Tippett D. C.. (2017). Important considerations in lesion-symptom mapping: illustrations from studies of word comprehension. Hum. Brain Mapp. 38, 2990–3000. 10.1002/hbm.23567 28317276 PMC5426992

[B81] Sokolov A. A. Gharabaghi A. Tatagiba M. S. Pavlova M. (2010). Cerebellar engagement in an action observation network. Cereb. Cortex 20, 486–491. 10.1093/cercor/bhp117 19546157

[B82] Stams G. J. Brugman D. Dekovic M. van Rosmalen L. van der Laan P. Gibbs J. C. (2006). The moral judgment of juvenile delinquents: a meta-analysis. J. Abnorm. Child Psychol. 34, 697–713. 10.1007/s10802-006-9056-5 17048108

[B83] Tamietto M. de Gelder B. (2010). Neural bases of the non-conscious perception of emotional signals. Nat. Rev. Neurosci. 11, 697–709. 10.1038/nrn2889 20811475

[B84] Terribilli D. Schaufelberger M. S. Duran F. L. Zanetti M. V. Curiati P. K. Menezes P. R.. (2011). Age-related gray matter volume changes in the brain during non-elderly adulthood. Neurobiol. Aging 32, 354–368. 10.1016/j.neurobiolaging.2009.02.008 19282066 PMC3004040

[B85] Torralva T. Roca M. Gleichgerrcht E. López P. Manes F. (2009). INECO Frontal Screening (IFS): a brief, sensitive, and specific tool to assess executive functions in dementia. J. Int. Neuropsychol. Soc. 15, 777–786. 10.1017/s1355617709990415 19635178

[B86] Uono S. Sato W. Kochiyama T. Sawada R. Kubota Y. Yoshimura S.. (2017). Neural substrates of the ability to recognize facial expressions: a voxel-based morphometry study. Soc. Cogn. Affect. Neurosci. 12, 487–495. 10.1093/scan/nsw142 27672176 PMC5390731

[B88] van den Bos W. Vahl P. Güroğlu B. van Nunspeet F. Colins O. Markus M.. (2014). Neural correlates of social decision-making in severely antisocial adolescents. Soc. Cogn. Affect. Neurosci. 9, 2059–2066. 10.1093/scan/nsu003 24493845 PMC4249473

[B87] van de Riet W. A. Grezes J. de Gelder B. (2009). Specific and common brain regions involved in the perception of faces and bodies and the representation of their emotional expressions. Soc. Neurosci. 4, 101–120. 10.1080/17470910701865367 19255912

[B89] Van den Stock J. Tamietto M. Sorger B. Pichon S. Grézes J. de Gelder B. (2011). Cortico-subcortical visual, somatosensory, and motor activations for perceiving dynamic whole-body emotional expressions with and without striate cortex (V1). Proc. Natl. Acad. Sci. U S A 108, 16188–16193. 10.1073/pnas.1107214108 21911384 PMC3182696

[B90] Van den Stock J. Vandenbulcke M. Sinke C. B. Goebel R. de Gelder B. (2014). How affective information from faces and scenes interacts in the brain. Soc. Cogn. Affect. Neurosci. 9, 1481–1488. 10.1093/scan/nst138 23956081 PMC4187263

[B91] Vilà-Balló A. Cunillera T. Rostan C. Hdez-Lafuente P. Fuentemilla L. Rodríguez-Fornells A. (2015). Neurophysiological correlates of cognitive flexibility and feedback processing in violent juvenile offenders. Brain Res. 1610, 98–109. 10.1016/j.brainres.2015.03.040 25839762

[B92] Vuilleumier P. Driver J. (2007). Modulation of visual processing by attention and emotion: windows on causal interactions between human brain regions. Philos. Trans. R. Soc. Lond. B Biol. Sci. 362, 837–855. 10.1098/rstb.2007.2092 17395574 PMC2430001

[B93] Vytal K. Hamann S. (2010). Neuroimaging support for discrete neural correlates of basic emotions: a voxel-based meta-analysis. J. Cogn. Neurosci. 22, 2864–2885. 10.1162/jocn.2009.21366 19929758

[B94] Wieser M. J. Keil A. (2014). Fearful faces heighten the cortical representation of contextual threat. Neuroimage 86, 317–325. 10.1016/j.neuroimage.2013.10.008 24125792 PMC4314112

[B95] Yue T. Pan W. Huang X. (2016). The relationship between trait positive empathy and brain structure: a voxel-based morphometry study. Neuroreport 27, 422–426. 10.1097/wnr.0000000000000557 26963166

[B96] Zhang S. Li C.-S. R. (2012). Functional connectivity mapping of the human precuneus by resting state fMRI. Neuroimage 59, 3548–3562. 10.1016/j.neuroimage.2011.11.023 22116037 PMC3288461

[B97] Zou Z. Meng H. Ma Z. Deng W. Du L. Wang H.. (2013). Executive functioning deficits and childhood trauma in juvenile violent offenders in China. Psychiatry Res. 207, 218–224. 10.1016/j.psychres.2012.09.013 23036491

